# Supplementary material for: UHPLC-Q-Exactive Orbitrap MS/MS-Based Untargeted Metabolomics and Molecular Networking Reveal the Differential Chemical Constituents of the Bulbs and Flowers of Fritillaria thunbergii
Source: Molecules. 2022 Oct 16;27(20):6944. doi: 10.3390/molecules27206944 (PMC9609367; doi:10.3390/molecules27206944)
Supplement: Supplementary file 1 [file molecules-27-06944-s001.zip › Table S3. The characteristic fragment ions of flavonoids used in chemical identification of present work.pdf]

Table S3. The characteristic fragment ions of flavonoids used in chemical identification of present work .

Compound	characteristic fragment ions in negative model [*m*/*z*]	Ref
Isorhamnetin derivatives	316.06 [Y_0]$^-$, 315.05 [Y_0-H]$^-$, 271.02 [M-H-CO2]$^-$, 300.03 [M-H-CH_3]$^-$, 255.03 [M-H-CH3-H_2O-CO]$^-$, 151.00 [$^{1,3}A^-$],	[1]
Quercetin derivatives	301.03 [Y_0]$^-$, 300.03 [Y_0-H]$^-$, 271.02 [M-H-CH_2O]$^-$, 255.03 [M-H-H_2O-CO]$^-$, 151.00 [$^{1,3}A^-$]	[2]
Kaempferol	286.05 [Y_0]$^-$, 285.04 [Y_0-H]$^-$, 255.03 [M-H-CH_2O]$^-$, 227.03. [M-H-CH_2O-CO]$^-$, 151.00 [$^{1,3}A^-$]	[2]
Luteolin	286.05 [Y_0]$^-$, 285.04 [Y_0-H]$^-$, 241.05 [M-H-CO2]$^-$, 217.05 [M-H-C3O2]$^-$, 175.04 [M-H-C3O2-C2H2O]	[3, 4]
Taxifolin	303.05 [Y_0-H]$^-$, 285.04 [M-H-CO2]$^-$, 125.02 [M-H-C2H2O]$^-$, 151.00 [$^{1,3}A^-$]	[5]

1. Du, L.-y.; Zhao, M.-T.; Qian, J.-H.; Jiang, D.-W.; et al.. The Metabolic Profiling of Isorhamnetin-3-O-Neohesperidoside Produced by Human Intestinal Flora Employing UPLC-Q-TOF/MS. Journal of Chromatographic Science. 2016, **55**(3), 243-250.
2. Schieber, A.; Berardini, N.; Carle, R. Identification of Flavonol and Xanthone Glycosides from Mango (Mangifera indica L. Cv. "Tommy Atkins") Peels by High-Performance Liquid Chromatography-Electrospray Ionization Mass Spectrometry. Journal of Agricultural and Food Chemistry. 2003, **51**(17), 5006-5011.
3. Śliwka-Kaszyńska, M.; Anusiewicz, I.; Skurski, P. The Mechanism of a Retro-Diels– Alder Fragmentation of Luteolin: Theoretical Studies Supported by Electrospray Ionization Tandem Mass Spectrometry Results. Molecules, 2022. **27**(3).
4. Brito, A.; Ramirez, J.-E.; Areche, C.; Sepulveda, B.; Simirgiotis, M.-J. HPLC-UV-MS Profiles of Phenolic Compounds and Antioxidant Activity of Fruits from Three Citrus Species Consumed in Northern Chile. Molecules. 2014, **19**(11), 17400-17421.
5. Hvattum, E., Determination of phenolic compounds in rose hip (Rosa canina) using liquid

chromatography coupled to electrospray ionisation tandem mass spectrometry and diode-array detection. Rapid Communications in Mass Spectrometry. 2002, **16**(7), 655-662.
