# Supplementary material for: UHPLC-Q-Exactive Orbitrap MS/MS-Based Untargeted Metabolomics and Molecular Networking Reveal the Differential Chemical Constituents of the Bulbs and Flowers of Fritillaria thunbergii
Source: Molecules. 2022 Oct 16;27(20):6944. doi: 10.3390/molecules27206944 (PMC9609367; doi:10.3390/molecules27206944)
Supplement: Supplementary file 1 [file molecules-27-06944-s001.zip › Table S4. The adduct ions or product ions of flavonoids.pdf]

Table S4. The other adduct ions of flavonoids included in the molecular networking

Peak ID	Node ID	RT (min)	Adduct	Observed *m/z* (Da)	Proposed Adduct ions information				
					Node ID	RT (min)	Observed *m/z* (Da)	*m/z* Error (ppm)	Proposed Adduct ions
H4	1919_N	15.89	[M-H]-	625.1403	2444	15.80	1349.2549		[2M+HSO4]-
					1970	15.93	693.1277		[M-H+HCOONa]-
					1960	15.85	745.0895		[M+NaSO4]-
					2048	15.82	865.0388		[M+2NaSO4]-
					2124	15.85	887.0207		[M+2NaSO4+Na-H]-
					2013	15.84	1251.287		[2M-H]-
					2707	15.84	1273.2685		[2M-H+Na]-
H7	1922_N	16.40	[M-H]-	303.0508	2057	16.40	607.1082		[2M-H]-
H8	1921_N	16.55	[M-H]-	609.1453	1998	16.57	677.1328		[M-H+HCOONa]-
					1971	16.53	729.0949		[M+NaSO4]-
					2222	16.53	1219.2971		[2M-H]-
H10	1944_N	16.77	[M-H]-	463.0877	2123	16.72	583.037		[M+NaSO4]-

H13	1924_N	17.23	[M-H]-	623.1622	1975	17.30	743.1105		[M+NaSO4]-
					2072	17.31	863.0594		[M+2NaSO4]-
					2002	17.31	691.1484		[M-H+HCOONa]-
